# 578. INO-4800 DNA Vaccine Induces Neutralizing Antibodies and T cell Activity Against Global SARS-CoV-2 Variants

**DOI:** 10.1093/ofid/ofab466.776

**Published:** 2021-12-04

**Authors:** Viviane M Andrade, Aaron Christensen-Quick, Joseph Agnes, Jared Tur, Charles C Reed, Richa Kalia, Idania Marrero, Dustin Elwood, Katherine Schultheis, Mansi Purwar, Emma Reuschel, Trevor McMullan, Patrick Pezzoli, Kimberly A Kraynyak, Albert Sylvester, Mammen P Mammen Jr., Pablo Tebas, J Joseph Kim, David Weiner, Trevor R F Smith, Stephanie Ramos, Laurent Humeau, Jean Boyer, Kate Broderick

**Affiliations:** 1 INOVIO Pharmaceuticals, Plymouth Meeting, Pennsylvania; 2 Inovio Pharmaceuticals, San Diego, California; 3 Wistar Institute, philadelphia, Pennsylvania; 4 INOVIO Pharamceuticals, San Diego, California; 5 Perelman School of Medicine, University of Pennsylvania, Philadelphia, Pennsylvania

## Abstract

**Background:**

Global surveillance has identified emerging SARS-CoV-2 variants of concern (VOC) associated with increased transmissibility, disease severity, and resistance to neutralization by current vaccines under emergency use authorization (EUA). Here we assessed cross-immune responses of INO-4800 vaccinated subjects against SARS-CoV-2 VOCs.

**Methods:**

We used a SARS-CoV-2 IgG ELISA and a pseudo neutralization assay to assess humoral responses, and an IFNγ ELISpot to measure cellular responses against SARS-CoV-2 VOC in subjects immunized with the DNA vaccine, INO-4800.

**Results:**

IgG binding titers were not impacted between wild-type (WT) and B.1.1.7 or B.1.351 variants. An average 1.9-fold reduction was observed for the P.1 variant in subjects tested at week 8 after receiving two doses of INO-4800 (Figure 1a). We performed a SARS-CoV-2 pseudovirus neutralization assay using sera collected from 13 subjects two weeks after administration of a third dose of either 0.5 mg, 1 mg, or 2 mg of INO-4800. Neutralization was detected against WT and the emerging variants in all samples tested. The mean ID_50_ titers for the WT, B.1.1.7, B.1.351 and P.1. were 643 (range: 70-729), 295 (range: 46-886), 105 (range: 25-309), and 664 (range: 25-2087), respectively. Compared to WT, there was a 2.1 and 6.9-fold reduction for B.1.1.7 and B.1.351, respectively, while there was no difference between WT and the P.1 variant (Figure 1b). Next, we compared cellular immune responses to WT and SARS-CoV-2 Spike variants elicited by INO-4800 vaccination. We observed similar cellular responses to WT (median = 82.2 IQR = 58.9-205.3), B.1.1.7 (79.4, IQR = 38.9- 179.7), B.1.351 (80, IQR = 40.0-208.6) and P.1 (78.3, IQR = 53.1-177.8) Spike peptides (Figure 2).

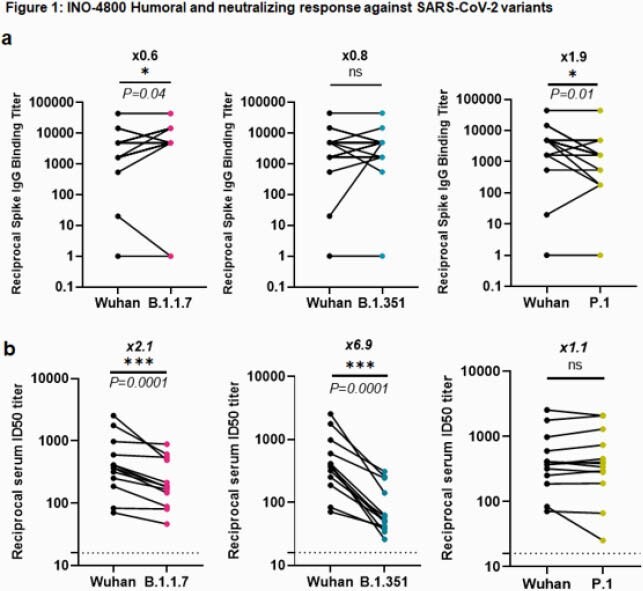

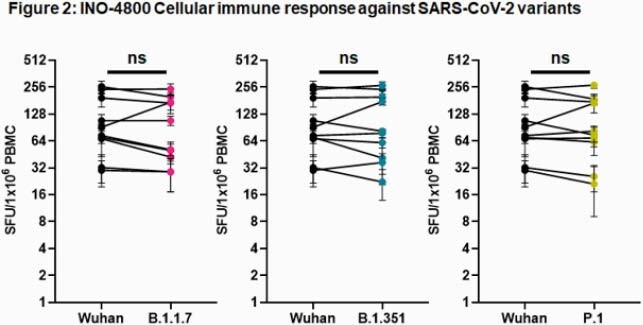

**Conclusion:**

INO-4800 vaccination induced neutralizing antibodies against all variants tested, with reduced levels detected against B.1.351. IFNγ T cell responses were fully maintained against all variants tested.

**Disclosures:**

**Viviane M. Andrade, PhD**, **Inovio Pharmaceuticals Inc.** (Employee) **Aaron Christensen-Quick, PhD**, **Inovio Pharmaceuticals, Inc** (Employee) **Joseph Agnes, PhD**, **Inovio** (Employee, Shareholder) **Jared Tur, PhD**, **Inovio** (Employee) **Charles C. Reed, PhD**, **Inovio Pharmaceuticals** (Employee, Shareholder) **Richa Kalia, MS**, **Inovio Pharmaceuticals** (Employee, Other Financial or Material Support, I have stock options with Inovio Pharmaceuticals as an employee.) **Idania Marrero, MD, PhD**, **Inovio Pharmaceuticals** (Employee, Shareholder) **Dustin Elwood, PhD**, **Inovio Pharmaceuticals** (Employee) **Katherine Schultheis, MSc**, **Inovio Pharmaceuticals** (Employee) **Emma Reuschel, PhD**, **Inovio Pharmaceuticals** (Employee) **Trevor McMullan, MSc**, **Inovio** (Shareholder) **Patrick Pezzoli, BS**, **Inovio** (Employee) **Kimberly A. Kraynyak, PhD**, **Inovio Pharmaceuticals** (Employee, Other Financial or Material Support, Stock options) **Albert Sylvester, MS**, **Inovio** (Employee, Shareholder) **Mammen P. Mammen Jr., MD**, **Inovio Pharmaceuticals** (Employee) **J Joseph Kim, PhD**, **Inovio** (Employee) **David Weiner, PhD**, **Inovio** (Board Member, Grant/Research Support, Shareholder, I serve on the SAB in addition to the above activities) **Trevor R. F. Smith, PhD**, **Inovio** (Employee, Shareholder) **Stephanie Ramos, PhD**, **Inovio Pharmaceuticals** (Employee) **Laurent Humeau, PhD**, **Inovio Pharmaceuticals** (Employee) **Jean Boyer, PhD**, **Inovio** (Employee) **Kate Broderick, PhD**, **Inovio** (Employee)

